# Do doctors who order more routine medical tests diagnose more cancers? A population‐based study from Ontario Canada

**DOI:** 10.1002/cam4.1925

**Published:** 2019-01-04

**Authors:** Stephen F. Hall, Colleen Webber, Patti A. Groome, Christopher M. Booth, Paul Nguyen, Yvonne DeWit

**Affiliations:** ^1^ Division of Cancer Care and Epidemiology, Department of Otolaryngology Queen’s University Kingston Ontario Canada; ^2^ Division of Cancer Care and Epidemiology, Department of Public Health Sciences Queen’s University Kingston Ontario Canada; ^3^ Division of Cancer Care and Epidemiology, Department of Oncology Queen’s University Kingston Ontario Canada; ^4^ ICES Queens Queen’s University Kingston Ontario Canada

**Keywords:** cancer overdiagnosis, imaging tests, laboratory tests, overuse, population‐based

## Abstract

**Background:**

The overuse of medical tests leads to higher costs, wasting of resources, and the potential for overdiagnosis of disease. This study was designed to determine whether the patients of family doctors who order more routine medical tests are diagnosed with more cancers.

**Method:**

A retrospective population‐based cross‐sectional study using administrative health care data in Ontario Canada. We investigated the ordering of 23 routine laboratories and imaging tests 2008‐20012 by 6849 Ontario family physicians on their 4.9 million rostered adult patients. We compared physicians’ test utilization and calculated case‐mix adjusted observed to expected (O:E) utilization ratios to categorize physicians as Typical, Higher or Lower testers. Age‐sex standardized rates (cases/10 000 patient years) and Rate Ratios were determined for cancers of the thyroid, prostate, breast, lymphoma, kidney, melanoma, uterus, ovary, lung, esophagus, and pancreas for each tester group.

**Results:**

There was wide variation in the use of the 23 tests by Ontario physicians. 26% and 24% of physicians were deemed Higher Testers for laboratory and imaging tests, while 41% and 38% were Typical Testers. The patients of higher test users were diagnosed with more cancers of thyroid (laboratory [RR 1.61, 95% CI 1.39‐1.87] and imaging [RR 2.08, 95% CI 0.88‐2.30]) and prostate (laboratory [RR 1.10, 95% CI 1.03‐1.18] and imaging [RR 1.05, 95% CI 1.00‐1.10]).

**Conclusion:**

There is a wide variation in the ordering of routine and common medical tests among Ontario family doctors. The patients of higher testers were diagnosed with more thyroid and prostate cancers.

## 1 BACKGROUND

Research on the overuse of medical tests in inpatient and outpatient medical practice^1^, ^2^, ^3^, ^4^ has lead to organizations such as Choosing Wisely^5^, ^6^ and Right Care^7^, ^8^ as well as the concepts around “Do not Do”^9^ and OverDiagnosis.^10^ The overuse of medical tests is simply the ordering of tests that are unnecessary and represents poor resource utilization. Due to many factors including the media, the public, and many physicians believe that more tests are better as they might uncover treatable disease^11^, ^12^; in clinical practice, physicians order routine tests or panels of routine tests for many reasons including defensive medicine.^13^

The indiscriminant or inappropriate use of routine tests will uncover subclinical malignant and nonmalignant disease in the general population but the term OverDiagnosis specifically refers to “disease that ultimately will not cause symptoms or early death.”^12^ In oncology, this refers to the identification of small, asymptomatic, or undetectable cancers that may never become symptomatic or life‐threatening.^14^, ^15^ Routine screening with tests such as prostate‐specific antigen, mammography, neck ultrasound, and computed tomography chest are all known to be associated with the OverDiagnosis of prostate,^16^ breast,^17^ thyroid,^18^, ^19^ and lung^20^ cancers, respectively. Other cancers that have been implicated in the OverDiagnosis story include uterus,^21^ kidney,^15^ melanoma,^22^ and esophagus^23^ all of which can be uncovered by specific tests that can be ordered or performed with minimal indications.

The objective of the study was to determine whether variations in the use of routine laboratory and imaging testing by physicians were associated with variations in the rates of cancer detection in their patients. The universal health insurance program and the availability of linked health care data on all patients at the Institute of Clinical Evaluative Sciences in the province of Ontario, Canada provides the opportunity to answer this question.

## 2 MATERIALS AND METHODS

### 2.1 Study design

This is a retrospective population‐based cohort study using electronic health care data from all 13 million residents in the Province of Ontario, Canada. Ontario has 14 health care regions (Local Health Integration Networks (LHIN)) subdivided into 97 subLHINs (3‐15 per LHIN). We utilized the data holdings of the Institute of Clinical Evaluative Sciences (ICES) including all health care‐related events for the patient population linked using an anonymous unique identifier for each person from 1/1/2008 to 12/31/2012.

### 2.2 Data sources

1. The Ontario Health Insurance Plan (OHIP) contains physician billing data including diagnostic tests (test type, date, referring doctor).
2. The Ontario Cancer Registry (OCR) is a passive cancer registry based on all pathology reports with a diagnosis of cancer in Ontario.
3. The Ontario Registered Person's Database provides demographic information on all residents of Ontario who are eligible for OHIP.
4. The ICES Physician Database (IPDB) provides demographic and specialization data on all active physicians in Ontario.
5. The Canadian Institute for Health Information data includes inpatient and outpatient hospitalization data on all patients in Canada.
6. The Office of the Ontario Registrar General provides information on vital status (date and cause of death) on residents of Ontario.
7. The Client Agency Program Enrolment (CAPE) is a registry created at ICES of patients enrolled (rostered) in primary care group practices and the physician to whom they are rostered. CAPE dataset identified the Usual Providers of Care (UPCs).

### 2.3 Study population

**Figure 1:**
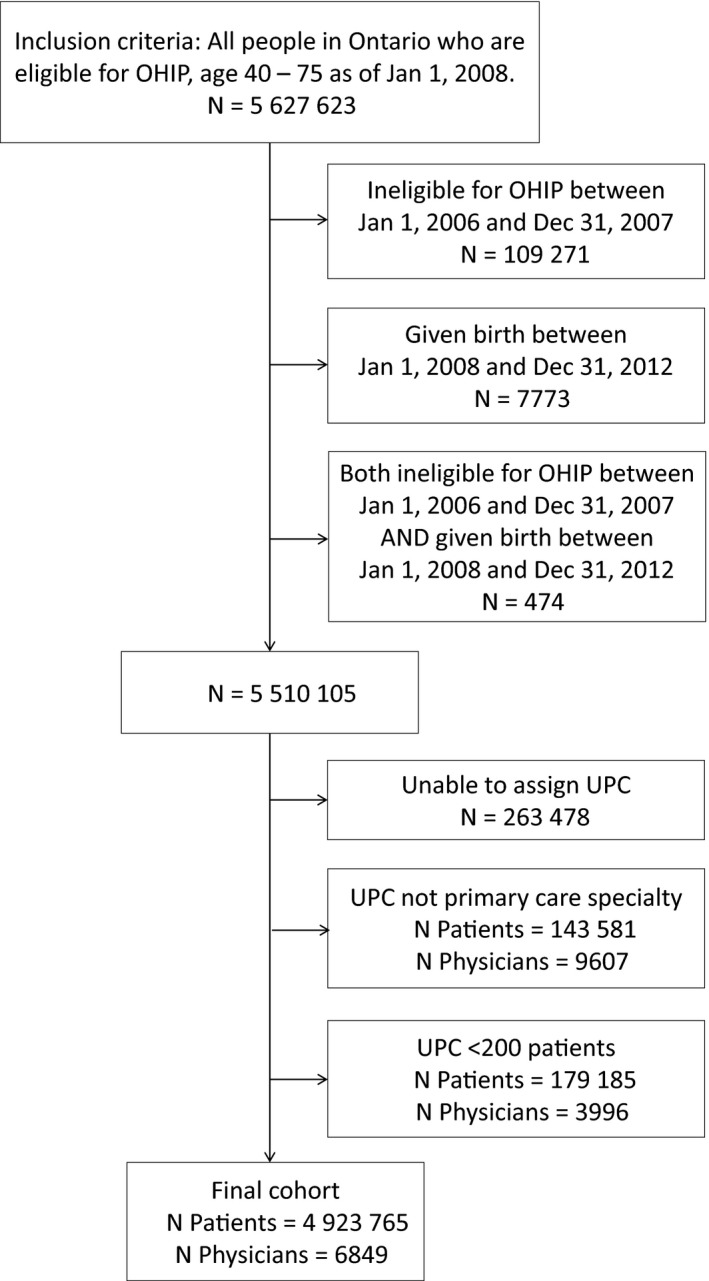
Flowchart of study population

The patient population included all adults age 40‐75 as of 1/1/2008 excluding 7773 women who gave birth during study period (as they would have had more tests) and patients without health care coverage (Figure 1). Patients who became ineligible for health care coverage after 2008 (109 271) were included until ineligibility. Approximately 3% of the population is not covered by the provincially funded universal health care (OHIP) and we have no access to information on them. These include transients, tourists, and those covered by Federal health insurance including active members of armed forces, indigenous persons living on reserves, inmates of federal prisons and some refugees.

In Ontario, Canada, most patients register or “sign on” to a single family physician in a group of family physicians who are then paid by the government by a mix of capitation payment, fee‐for‐service, and incentive fees. Although rostered patients can and do go to the ER and to “walk‐in” clinics, they usually are seen by their registering family doctor or another provider in the same group. They do not attend other family doctors groups. We identified the Usual Providers of Care (UPCs) caring for the study population 2008‐2012. Physicians without complete information in the ICES Physician Database (age, sex, practice type), those with small practices (<200 rostered patients) (3996), and those who were not primarily involved with primary care (9607) were excluded. Also, excluded were 263 478 patients who were not rostered to a study UPC or rostered at all as we wanted to look at the tests and cancers of patients who were going to their regular doctor if they had one.

### 2.4 Patient characteristics

1. Area‐level socioeconomic status (SES) of location‐specific income quintiles was based on Statistics Canada 2006 census data matched to postal codes providing data at level of enumeration units (neighborhoods)
2. Comorbidity was estimated using the Elixhauser Index^24^, ^25^, ^26^ based on hospital discharge data with look back of 2 years (1/1/2006 to 12/31/2007). Elixhauser created a summative scale over 31 domains for administrative data and we used the cut‐points of 0, 1, 2, >2 with greater comorbidity creating higher scores.^27^, ^28^, ^29^
3. Time on study was the number of consecutive patient days from 1/1/2008 to the earliest date of death, loss OHIP eligibility or end of study period (12/31/2012).
4. Rurality was based on the Statistics Canada Postal Code Conversion File from the 2006 census and reported by Metropolitan Influence Zones (MIZ).

### 2.5 UPC characteristics

Age, sex, years in practice and practice type (in 2008).

### 2.6 Study cancers and patient cancer rates

The outcome of interest was a new diagnosis of cancer. Based on the potential for OverDiagnosis, 11 cancers were selected including thyroid and prostate (definite) and breast, Non‐Hodgkin's Lymphoma, kidney, melanoma, uterus (possible). Cancers of ovary, lung, esophagus, and pancreas were included as controls. We assumed that almost all the aggressive cancers would progress within our time frame, would become clinically apparent and therefore be diagnosed at the same rates regardless of rates of medical tests. We calculated age‐sex standardized cancer incidence rates for each cancer (cases/10 000 patient years).

### 2.7 Test selection and utilization

A panel of common laboratory and imaging tests was developed based on meetings with 5 local family physicians (academic and community‐based) and a general internist. Tests had to be common, routinely ordered, potentially overused as screening tests and span a wide variety of clinical indications. Cancer‐specific tests with either formal cancer screening programs (fecal occult blood, mammography) or informal cancer screening (prostate‐specific antigen, Papanicolaou test) were excluded. A total of 27 tests were selected (see Table 3).

This study was about the impact of the variation in the rates of use of tests and therefore to establish a relationship between the rates of test use and rates of cancer diagnoses, tests had to demonstrate variation in use. To compare test rates, we first calculated the total # of each test performed on the population of each subLHIN and then age and sex standardized each subLHIN's test rates (# of tests/10 000 person‐years) to the entire study population. We assessed test rate variation by comparing test rates across the small geographic subLHINs using the Systematic Component of Variation (SCV)^30^, ^31^ for each test for each subLHIN. According to Appleby et al,^30^ the SCV is the appropriate measurement of variation for research in this setting, SCVs greater than 3 are likely to be due largely to differences in practice style or medical discretion, SCVs up to 10 are considered high variation and SCVs >10 very high variation.

### 2.8 Physician test utilization

We calculated observed‐to‐expected (O/E) ratios for each UPC for each test using indirect standardization, using the entire study population as the standard population. Indirect standardization was used as we were investigating variations in test utilization across physicians relative to the whole study population; indirect standardization allowed us to explore these variations while removing the confounding effect of patient age and sex. Observed (actual) counts were the # of tests in study period of those patients in the practice of each UPC for their patient years. The expected count was the # tests a UPC might order based on his/hers case mix if his/her test utilization was identical to that observed in the entire study population. This count was done by initially calculating a rate for each of 14 age/sex strata (ie, male 40‐44, female 70‐75 etc) across Ontario for each test for the total patient years in each strata. An expected count was estimated for each strata, with the total sum being the expected count for each UPC for each test. To create composite O/E ratios for all the laboratory tests and all the imaging tests for each UPC, all of their patients observed 13 laboratory test and 10 imaging test counts were summed. Then, the expected counts for the total laboratory and imaging tests for all those patients were generated using indirect patient age‐sex standardization for each UPC. The combined test O/E ratios are based on the ratio of the combined total observed and total expected test counts (13 laboratory tests or 10 imaging test).

### 2.9 Physician testers

To assess over and underuse of tests by UPCs, we created 6 levels of physician testers based on the O/E ratios of all the UPCs for the combined laboratory and for the combined imaging tests. Six levels were the smallest number of groups that could provide estimates for lower and typical testers as well as the opportunity to assess dose‐response.. Typical testers were defined as an O/E ratio of 0.75‐1.25. We selected <0.5 for strong lower testers, 0.5‐0.75 for mild lower testers, 1.25‐1.5 for mild higher testers, 1.5‐2.0 for moderate higher testers and >2.0 for strong higher testers. The thresholds for all tester groups were defined a priori and were based on our assumption that a 25% and especially a 50% increase or decrease in the ordering of routine tests would be of clinical significance.

### 2.10 UPC testers vs cancer incidence

The relationship between tester groups and cancer incidence rates was evaluated by examining age‐sex standardized incidence rates and risk ratios.

### 2.11 Statistical analysis

All statistical tests were two‐sided with significance of 0.05. Poisson regression models were used to control for effects of patient and UPC variables on the risk of cancer diagnoses. Patients with missing or incomplete variable data were not included in the modelling.

## 3 RESULTS

### 3.1 Study population and cancers

The study populations included 4 923 765 residents of Ontario and 6849 UPCs (Table 1). There was no difference in patient age or comorbidity across the tester groups. Males, rural patients, and lower SES tended toward few tests. At least one of the 11 study cancers was diagnosed in 139 248 patients during 2008‐2012 (Table 2). Prostate, breast, and lung had the greatest number of new cases and the highest rates of new cases. Ovary, pancreas, and esophagus were the least common new diagnoses.

**Table 1:** Patient Characteristics (n = 4,923,765) (** due to missing or incorrect address/postal code or classifications, there are missing data in some cells)

Variable	Total	Laboratory tests			Imaging tests		
		All lower testers	Typical testers	All higher testers	All lower testers	Typical testers	All higher testers
Age (mean)	54.4 y	54.5 y	54.3 y	54.4 y	54.3 y	54.5 y	54.3 y
Sex							
Male, %	48.28	50.91	47.67	46.41	51.55	47.04	45.47
Elixhauser comorbidity index, %							
0	93.40	93.1	93.56	93.48	93.56	93.32	93.30
1	3.81	3.95	3.73	3.78	3.64	3.89	3.94
2	1.51	1.57	1.47	1.49	1.50	1.51	1.52
>2	1.28	1.37	1.24	1.24	1.30	1.28	1.24
Neighborhood income quintiles**, %							
1 (Lowest)	17.57	18.76	17.10	17.02	18.73	16.75	17.16
2	19.42	19.27	19.26	19.82	19.85	19.18	19.17
3	19.88	19.48	19.77	20.47	19.63	19.79	20.38
4	21.07	20.32	21.33	21.45	20.35	21.28	21.77
5 (Highest)	21.77	21.75	22.27	21.01	21.12	22.71	21.27
NA/Unknown	0.30	0.42	0.26	0.23	0.33	0.30	0.26
Rurality**, %							
Urban	87.20	80.42	88.64	92.24	85.90	86.45	90.25
Strong MIZ	5.53	6.40	5.76	4.25	5.77	5.46	5.31
Moderate MIZ	4.84	7.49	4.31	2.81	5.27	5.60	3.03
Weak/No MIZ	2.42	5.67	1.28	0.70	3.04	2.48	1.41
NA/Unknown	0.01	0.02	0.01	0.01	0.02	0.01	0.01

**Table 2:** The 139,248 new cancers diagnosed in the study population between Jan 1, 2008 and Dec 31, 2012

Cancer	Total no. of diagnoses	Total no. of person‐years (PY)	Rate (no. of diagnoses per 10 000 PY)
Thyroid	7823	24 085 903.58	3.25
Breast	30 240	12 439 627.67	24.31
Ovarian	3629	12 502 365.33	2.90
Uterus	7928	12 492 233.33	6.35
Prostate	34 072	11 512 565.67	29.60
Esophagus	2390	24 101 589.25	0.99
Kidney	7157	24 089 698.25	2.97
Lung	26 813	24 076 212.58	11.14
Melanoma	7671	24 086 996.50	3.19
Pancreas	4577	24 100 576.75	1.90
Non‐Hodgkin's Lymphoma	8934	24 086 875.33	3.71
≥1 Above Cancers	139 248		

### 3.2 Test utilization

There were large differences in overall rates (tests/100 patient years) and in the variations in the rates for the selected tests across the 97 subLHINs (data not included). Chest X‐ray and Abdominal Ultrasound had the highest median rates and Limb CT, Neck Ultrasound and Spine CT had the highest variations in rates for imaging tests. Serum cholesterol/triglycerides, electrolytes, and Glutamate Pyruvate Transaminase had the highest median rates, and Ferritin, Vitamin B12, and Alkaline Phosphatase the highest variation in rates for the laboratory group.

We rejected 4 tests with SVC less than 3 (X‐ray of chest, foot and knee, and pelvic CT scans). A total of 22 tests with SCV range between 5.9 and 38.9 were selected for evaluation. Abdominal Ultrasound (SCV = 2.4) was retained as it was included in previous work.^18^

### 3.3 O/E ratios for UPCs

Table 3 lists the mean, maximum, and Inter‐quartile range (IQR) values of the O/E ratios for all UPCs for the selected tests. The laboratory tests with the highest O/E ratios were Anti‐Nuclear Antibody, Erythrocyte Sedimentation Rate and Creatinine. Similarly, the imaging tests with the highest ratios were abdominal X‐ray, neck ultrasound and limb ultrasound.

**Table 3:** The Observed/Expected Ratios for the laboratory and imaging tests

Test group	Test	Mean	Max	Inter quartile range (IQR)
Lab	Alkaline phosphatase	0.947	8.500	0.241‐1.465
	Antinuclear antibody test	0.979	45.566	0.243‐1.209
	Complete blood count	0.966	5.742	0.662‐1.264
	Cholesterol/Triglycerides	0.960	4.538	0.668‐1.251
	Creatinine	0.964	11.565	0.647‐1.262
	Electrolytes	0.977	5.833	0.417‐1.399
	Erythrocyte sedimentation rate	0.963	25.196	0.243‐1.113
	Ferritin	0.971	9.304	0.259‐1.517
	Glycosylated hemoglobin	0.945	6.917	0.471‐1.282
	High‐density lipoprotein	0.960	4.555	0.666‐1.252
	Serum glutamate pyruvate transaminase	0.973	6.150	0.504‐1.296
	Thyroid‐stimulating hormone	0.963	5.806	0.572‐1.323
	Vitamin B12	0.971	9.936	0.262‐1.527
Imaging	Abdominal CT	0.962	8.589	0.382‐1.305
	Abdominal ultrasound	0.955	16.380	0.441‐1.214
	Abdominal X‐ray	0.992	46.193	0.193‐1.230
	Bone mineral density	0.957	5.980	0.504‐1.312
	Bone Scan	0.920	17.078	0.233‐1.199
	Carotid and/or artery ultrasound	0.965	15.804	0.352‐1.272
	Head CT	0.969	7.181	0.257‐1.403
	Limb ultrasound	0.958	20.094	0.342‐1.272
	Neck ultrasound	0.944	39.435	0.331‐1.072
	Spine CT	0.934	14.653	0.137‐1.238

### 3.4 UPC tester groups

**Figure 2:**
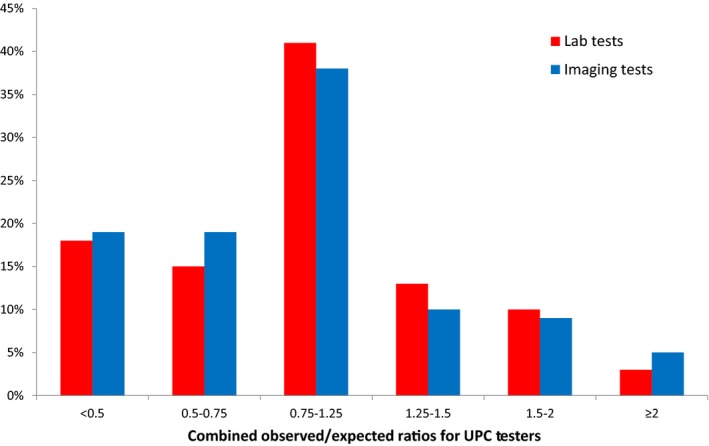
The distributions of the UPC test users based on O/E ratios for both imaging and laboratory tests

The distribution of the UPC tester groups (Strong Lower Testers, Mild Lower Testers, Typical Testers, Mild Higher Testers, Moderate Higher Testers, Strong Higher Testers) based on the O/E ratios for both the laboratory and imaging test groups are presented on Figure 2. 26% and 24% of the UPCs were higher testers for laboratory and imaging, respectively. 33% and 38% of the UPCs were lower testers for laboratory and imaging tests. Overall, UPCs in the Typical Tester group ordered an average of 18,688 laboratory and 669 imaging tests in 2008‐2012. The Higher Testers ordered on average 59% (29,800) more laboratory and 80% (1,207) more imaging tests than the Typical Testers. The Lower Testers ordered on average 61% (7,259) fewer laboratory and 57% fewer imaging tests. The distributions for 3 Higher Tester groups for both laboratory and imaging tests were stable over time comparing the cohorts of 1/1/08‐31/5/10 to 1/6/10 to 31/12/12 (data not included).

### 3.5 Cancer rates by UPC tester groups

**Figure 3:**
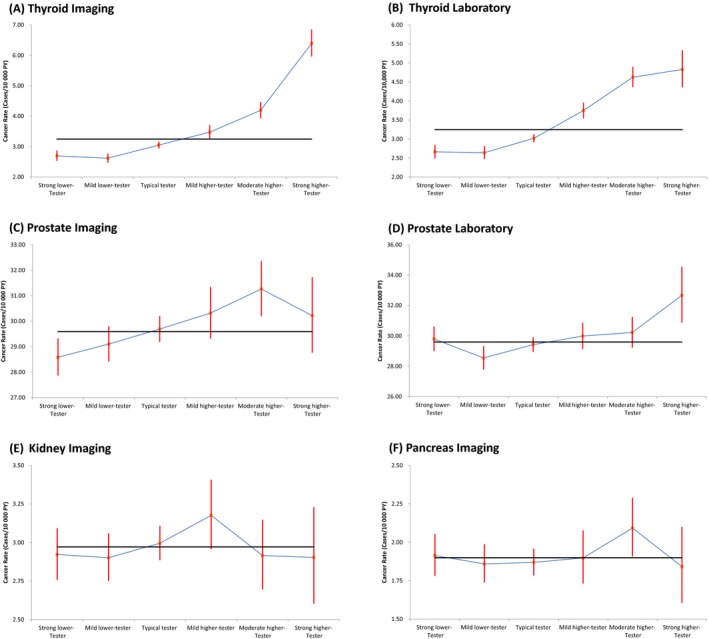
The age/sex standardized rates (cases/10 000 patient years) for the UPC laboratory and imaging tester groups. The horizontal line is the overall cancer rate for the study population

The age‐sex standardized rates (cases/10 000 patient years) for our study population of select cancers by the UPC tester groups are presented on Figure 3. Only thyroid cancer had significantly higher cancer incidence rates in all higher testers (average rates of 4.2/10 000 patient years and 4.4/10 000 patient years among all higher testers of laboratory and imaging tests vs. overall rate of 3.2/10 000 patient years) and lower cancer incidence rates in all lower testers (average rates of 2.7/10 000 patient years and 2.7/10 000 patient years among all lower testers of laboratory and imaging tests vs. overall rate of 3.2/10 000 patient years) for both the laboratory and imaging groups of tests (Figure 3A,B). Prostate cancer demonstrated increased incidence rates with some of higher tester groups for both laboratory and imaging (rates of 32.7/10 000 patient years among strong higher tester of laboratory tests and 31.3/10 000 patient years among moderate higher tester of imaging tests vs. overall rate of 29.6/10 000 patient years) and decreased incidence rates with some of the lower tester laboratory and imaging groups (rates of 28.5/10 000 patient years among moderate lower tester of laboratory tests and 28.6/10 000 patient years among strong lower tester of imaging tests vs. overall rate of 29.6/10 000 patient years) (Figure 3C,D). Pancreas cancer had higher incidence in the moderate higher imaging testers group of UPCs (Figure 3F). Of the remaining 8 study cancers (breast, ovary, lung, esophagus, uterus, kidney, melanoma, and NHL), none had statistically significant increases in rates with increasing testing noting that breast, uterus, kidney (Figure 3E) and Non‐Hodgkins Lymphoma demonstrated nonstatistically significant increases in rates with some higher imaging user groups.

### 3.6 Rate Ratios (RRs) of a cancer diagnosis by UPC tester groups

**Figure 4:**
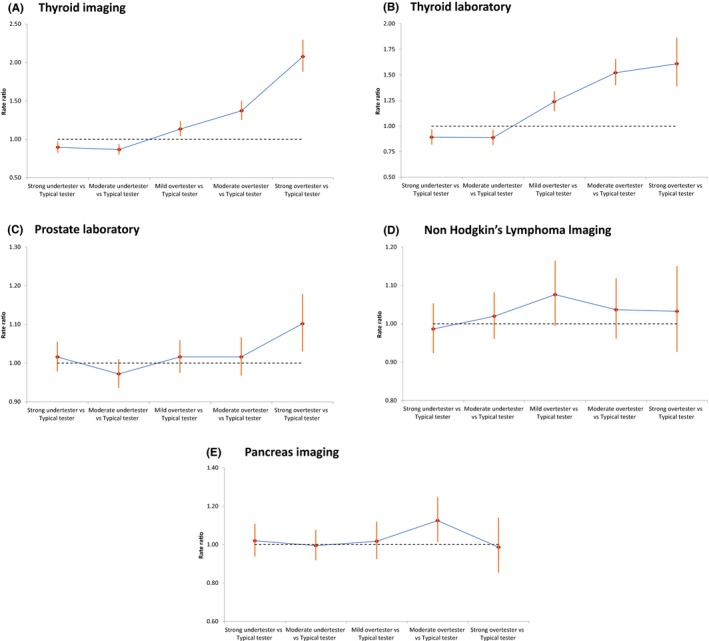
The Rate Ratios (RRs) for cancer risk (adjusted for patient age, patient sex, patient comorbidity, UPC age, UPC sex). Typical testers are the control group in the regression model

The cancer incidence RRs for UPC test groups when controlling for patient factors (age, sex, and comorbidity) and physician factors (age and sex) are presented for select cancers on Figure 4. The overall results are reported in Table S1. The reference group is the Typical Testers. Similar to the results of rates on Figure 3, thyroid (Figure 4A,B) was the only cancer to be diagnosed more by all higher testers and less by all lower testers for both laboratory and imaging tests. The only other cancers to have significant increases with increasing use of tests were prostate (Figure 4C) and pancreas cancers (Figure 4E). Of the remaining 8 study cancers (breast, ovary, lung, esophagus, uterus, kidney, melanoma, and NHL), none had statistically significant risk (ie, OR = 1.0) with increasing testing noting that breast, uterus and Non‐Hodgkins Lymphoma (Figure 4D), demonstrated nonsignificant increases with more tests. Adjustment for additional physician factors (years in practice) or patient factors (income, rurality, and deprivation) or using sex‐stratified analyses changed the levels of significance but not the significant results.

## 4 DISCUSSION

The objective of this study was to determine whether variations in the overall rates of ordering routine laboratory and imaging testing by physicians were associated with variations in the rates of cancer detection in their patients. Our method included a variety of selected tests noting that patients will have had these tests performed for legitimate reasons, for unrelated but important reasons, and for no reason aside from screening. There is no information on what the correct, ideal, or appropriate rates of our selected tests might be across a population and we based our classification of testers on the test rates of the average or Typical Testers. Our results are not meant to reflect ideal testing physicians or ideal treating physicians. We found that 24% and 26% of physicians were higher laboratory and imaging testers, respectively, compared to their peers and as expected that thyroid cancer and prostate cancers were diagnosed more by higher laboratory testers and higher imaging testers. These findings are consistent with the literature on screening, OverDiagnosis and subsequent overtreatment for both thyroid and prostate cancer. We also found that the patients of doctors who were lower testers were diagnosed with fewer thyroid cancers. We did not expect to and did not find a relationship between higher testing and cancers of lung, esophagus and ovary. Nonstatistically significant trends were seen in other cancers implicated in the OverDiagnosis literature (breast, uterus, kidney) suggesting that the overuse of tests, subclinical disease, and a proportion of indolent cases along with the potential for overtreatment might be part of the stories in those cancers. Unexpectedly, we found that the patients of doctors who were lower testers were diagnosed with more ovary, lung, esophagus and kidney cancers; reasons for this (perhaps social reasons by patients) would be speculative and are outside the scope of this study.

Our finding that rates of pancreas cancer are related to rates of routine testing (Figures 3F and 4D) was unexpected. Pancreas cancer was diagnosed more often by the Moderate higher imaging tester group and had consistent marginal results throughout all the analyses of imaging tests. The incidence of pancreas cancer is not changing in Canada^32^ and mortality is slowly declining.^33^ In the United States, however, the incidence is slowly rising and mortality is flat^34^ which is the typical pattern of an overdiagnosed cancer.^15^ There appears to be a role for screening the <10% of patients with a family history and there are recognized premalignant lesions (pancreatic intraepithelial neoplasia and intraductal papillary mucinous neoplasm) that potentially could be picked up by high testers. The other surprise cancer was Non‐Hodgkins Lymphoma. Although the rates for Non‐Hodgkins Lymphoma never achieved statistical significance, evidence of a marginal increase in rates with higher users was a consistent finding throughout our analysis unlike all the other cancers we tested. The incidence of Non‐Hodgkins Lymphoma is declining and survival is improving in Canada and the United States.

We made a number of assumptions for this study. We, for example, assumed that doctors who ordered more routine tests (perhaps some inappropriate or unnecessary) on behalf of their patients would also order more disease‐specific tests (or cancer‐specific tests) as screening tests. This is a reasonable assumption that cannot be proven as the data linking family doctors to breast, cervix and colon screening testing is incomplete. We assumed the rates of UPC tests from 2008 to 2012 would reflect practice for the few years before 2008.

The complete linked dataset for a very large study population and their doctors is the strength of this study; however, there are potential limitations. First, we chose an informal test selection process instead of a more rigorous modified Delphi process. We felt this was appropriate for our question as were looking for common tests ordered by family physicians on a background of Choosing Wisely and common sense. It is unlikely that different tests would have been selected by a more time consuming and expensive process. Second, in the absence of any relevant literature on the comparative clinical impact of test overuse and underuse, the authors assumed that the cutpoints of 25%, 50% or 100% more tests had clinical relevance. We did post hoc explore other statistical options such as standard deviations and there was no difference in the overall results (data not included). Third, we could not analyze cancer rates by Stage or extent of disease as “stage data” was not completely or reliability available for the 11 sites during the study time. A future study with “stage data” might compare the overuse of tests and early stage disease. Fourth, we could not assess the indications for the tests including patient wishes. Fifth, there were missing patients including 263 478 who could not be assigned to a UPC and women who gave birth during the study period (7773) as they would have had more routine tests. Patients who became ineligible for health care coverage after 2008 (109 271) were included until ineligibility. We excluded over 12,000 family physicians including those without information in the ICES dataset, UPCs with small practices and UPCs who were not involved in full‐time primary care noting that there is no reason to suspect that similar doctors with similar patients would not have similar testing behavior within our health care system. Sixth, our results are specific to the 23 tests, the 11 common cancers and the universal health care system in Ontario and may not be generalizable to other tests, other cancers and other health care funding systems. Finally, in Ontario, routine laboratory tests done on outpatients at hospitals are not billed to OHIP and therefore do not appear in our datasets. We therefore may have underestimated the rates of routine laboratory tests but this represents only 5% of tests^35^ and is unlikely to influence our results.

## 5 CONCLUSION

Due to the wide variation in the ordering of common and routine laboratory and imaging tests, family doctors in Ontario Canada could be classified into Typical, Higher and Lower testers. As predicted by the literature on OverDiagnosis, the patients of physicians who were Higher testers were diagnosed more often with thyroid and prostate cancers. The overuse of medical tests in a health care system leads to the OverDiagnosis with downstream implications of overtreatment and increased costs. Mechanisms to address and correct overuse of tests through education would result in reductions in morbidity and cost.

## CONFLICT OF INTEREST

The authors have no conflicts of interest.
