# Supplementary material for: Do doctors who order more routine medical tests diagnose more cancers? A population‐based study from Ontario Canada
Source: Cancer Med. 2019 Jan 4;8(2):850–9. doi: 10.1002/cam4.1925 (PMC6382726; doi:10.1002/cam4.1925)
Supplement: Supplementary file 1 [file CAM4-8-850-s001.docx]

**Table 4**: Rate Ratios for a new diagnosis of cancer by UPC groups (Typical Tester is the control group). Red font indicates a significant increase in rate of diagnosis (p<0.05)

**Table 4a: Laboratory tests**

| **Cancer** | **Strong**  **Lower-tester** | **Moderate**  **Lower-tester** |  | **Mild**  **Higher-tester** | **Moderate**  **Higher-tester** | **Strong**  **Higher-tester** |
| --- | --- | --- | --- | --- | --- | --- |
| Thyroid | 0.89 (0.82-0.97) | 0.89 (0.82-0.96) |  | 1.24 (1.14-1.34) | 1.52 (1.40-1.66) | 1.61 (1.39-1.87) |
| Breast | 1.03 (0.99-1.07) | 1.04 (1.00-1.08) |  | 1.01 (0.98-1.05) | 1.00 (0.96-1.04) | 1.03 (0.96-1.09) |
| Ovarian | 1.09 (0.99-1.21) | 0.87 (0.79-0.97) |  | 1.03 (0.93-1.13) | 0.89 (0.79-1.00) | 1.00 (0.82-1.22) |
| Uterus | 1.06 (1.00-1.14) | 1.05 (0.98-1.12) |  | 1.00 (0.93-1.07) | 1.02 (0.95-1.11) | 0.98 (0.87-1.11) |
| Prostate | 1.02 (0.98-1.06) | 0.97 (0.94-1.01) |  | 1.02 (0.97-1.06) | 1.02 (0.97-1.07) | 1.10 (1.03-1.18) |
| Esophagus | 1.22 (1.09-1.36) | 1.09 (0.97-1.22) |  | 0.88 (0.77-1.00) | 0.72 (0.61-0.85) | 0.87 (0.68-1.12) |
| Kidney | 1.08 (1.00-1.15) | 1.01 (0.95-1.09) |  | 0.99 (0.91-1.06) | 0.93 (0.86-1.02) | 0.98 (0.86-1.12) |
| Lung | 1.13 (1.09-1.18) | 1.03 (0.98-1.07) |  | 0.93 (0.89-0.97) | 0.92 (0.87-0.97) | 0.85 (0.78-0.94) |
| Melanoma | 1.04 (0.96-1.11) | 1.05 (0.97-1.13) |  | 0.88 (0.81-0.95) | 0.79 (0.72-0.87) | 0.81 (0.68-0.97) |
| Pancreas | 1.05 (0.96-1.14) | 1.07 (0.98-1.16) |  | 0.96 (0.87-1.05) | 1.04 (0.95-1.15) | 1.13 (0.97-1.32) |
| Non-Hodgkin's Lymphoma | 1.02 (0.95-1.09) | 1.03 (0.97-1.10) |  | 0.91 (0.85-0.98) | 1.02 (0.95-1.11) | 0.97 (0.86-1.10) |

**Table 4b: Imaging tests**

| **Cancer** | **Strong**  **Lower-tester** | **Moderate**  **Lower-tester** |  | **Mild**  **Higher-tester** | **Moderate**  **Higher-tester** | **Strong**  **Higher-tester** |
| --- | --- | --- | --- | --- | --- | --- |
| Thyroid | 0.90 (0.82-0.97) | 0.87 (0.80-0.94) |  | 1.13 (1.04-1.24) | 1.37 (1.25-1.50) | 2.08 (1.88-2.30) |
| Breast | 0.98 (0.94-1.01) | 0.98 (0.95-1.01) |  | 0.99 (0.95-1.03) | 1.02 (0.98-1.06) | 1.01 (0.96-1.06) |
| Ovarian | 1.04 (0.94-1.15) | 1.06 (0.97-1.16) |  | 1.00 (0.89-1.12) | 1.00 (0.90-1.12) | 1.01 (0.86-1.18) |
| Uterus | 1.00 (0.94-1.07) | 0.99 (0.93-1.06) |  | 0.99 (0.92-1.08) | 1.03 (0.96-1.11) | 1.01 (0.91-1.11) |
| Prostate | 0.96 (0.93-1.00) | 0.98 (0.95-1.02) |  | 1.02 (0.97-1.07) | 1.05 (1.00-1.10) | 1.01 (0.94-1.08) |
| Esophagus | 1.12 (0.99-1.25) | 1.00 (0.89-1.11) |  | 0.87 (0.75-1.01) | 1.02 (0.89-1.17) | 0.69 (0.54-0.88) |
| Kidney | 0.98 (0.91-1.05) | 0.97 (0.91-1.04) |  | 1.06 (0.98-1.15) | 0.97 (0.89-1.07) | 0.96 (0.86-1.08) |
| Lung | 1.06 (1.01-1.10) | 1.00 (0.96-1.04) |  | 1.00 (0.95-1.06) | 0.98 (0.92-1.03) | 0.79 (0.73-0.86) |
| Melanoma | 0.87 (0.81-0.94) | 0.96 (0.89-1.03) |  | 0.93 (0.84-1.01) | 0.90 (0.82-0.99) | 0.67 (0.58-0.78) |
| Pancreas | 1.02 (0.94-1.11) | 0.99 (0.92-1.08) |  | 1.02 (0.92-1.12) | 1.12 (1.01-1.25) | 0.99 (0.85-1.14) |
| Non-Hodgkin's Lymphoma | 0.99 (0.92-1.05) | 1.02 (0.96-1.08) |  | 1.08 (0.99-1.16) | 1.04 (0.96-1.12) | 1.03 (0.93-1.15) |
